# Cross calibration of ^123^I-*meta*-iodobenzylguanidine heart-to-mediastinum ratio with D-SPECT planogram and Anger camera

**DOI:** 10.1007/s12149-017-1191-2

**Published:** 2017-07-08

**Authors:** Kenichi Nakajima, Koichi Okuda, Kunihiko Yokoyama, Tatsuya Yoneyama, Shiro Tsuji, Hiroyuki Oda, Mitsuhiro Yoshita, Koji Kubota

**Affiliations:** 1 Department of Nuclear Medicine, Kanazawa University, 13-1 Takara-machi, Kanazawa, 920-8641 Japan; 2 PET Imaging Center, Public Central Hospital of Matto Ishikawa, Hakusan, Japan; 3 Department of Physics, Kanazawa Medical University, Uchinada, Kahoku, Japan; 4 Department of Cardiology, Public Central Hospital of Matto Ishikawa, Hakusan, Japan; 5 Department of Neurology, Hokuriku National Hospital, Nanto, Japan

**Keywords:** Heart-to-mediastinum ratio, Quantitation, Standardization, Sympathetic imaging, Conversion coefficient

## Abstract

**Background:**

Cardiac ^123^I-*meta*-iodobenzylguanidine (MIBG) uptake is quantified using the heart-to-mediastinum ratio (HMR) with an Anger camera. The relationship between HMR determined using D-SPECT with a cadmium–zinc–telluride detector and an Anger camera is not fully understood. Therefore, the present study aimed to define this relationship using images derived from a phantom and from patients.

**Methods:**

Cross-calibration phantom studies using an Anger camera with a low-energy high-resolution (LEHR) collimator and D-SPECT, and clinical ^123^I-MIBG studies proceeded in 40 consecutive patients (80 studies). In the phantom study, a conversion coefficient (CC) was defined based on phantom experiments and applied to the Anger camera and the D-SPECT detector. The HMR was calculated using anterior images with the Anger camera and anterior planograms with D-SPECT. First, the HMR from D-SPECT was cross-calibrated to the Anger camera, and then, the HMR from both cameras were converted to the medium-energy general-purpose collimator condition (CC 0.88; ME88 condition). The relationship between HMR and corrected and uncorrected methods was examined. A ^123^I-MIBG washout rate was calculated using both methods with and without background subtraction.

**Results:**

Based on the phantom experiments, the CC of the Anger camera with an LEHR collimator and of D-SPECT using an anterior planogram was 0.55 and 0.63, respectively. The original HMR from the Anger camera and D-SPECT was 1.76 ± 0.42 and 1.86 ± 0.55, respectively (*p* < 0.0001). After D-SPECT HMR was converted to the Anger camera condition, the corrected D-SPECT HMR became comparable to the values under the Anger camera condition (1.75 ± 0.48, p = n. s.). When the HMR measured using the two cameras were converted under the ME88 condition, the average standardized HMR from the Anger camera and D-SPECT became comparable (2.21 ± 0.65 vs. 2.20 ± 0.75, p = n. s.). After standardization to the ME88 condition, a systematic difference in the linear regression lines disappeared, and the HMR from both the Anger (StdHMR_Anger_) and D-SPECT (StdHMR_DSPECT_) became comparable. Additional correction using a regression line further improved the relationship between both HMR [StdHMR_DSPECT_ = 0.09 + 0.98 × StdHMR_Anger_ (*R* ^2^ = 0.91)]. The washout rate closely correlated with and without background correction between both methods (*R* ^2^ = 0.83 and 0.65, respectively).

**Conclusion:**

The phantom-based conversion method is applicable to D-SPECT and enables the common application of HMR irrespective of D-SPECT and the Anger camera.

## Introduction

Several multicenter studies and meta-analysis in Europe, the USA and Japan have indicated the value of sympathetic innervation imaging using ^123^I-*meta*-iodobenzylguanidine (MIBG) for patients with heart failure (HF) [1–5]. The Clinical Practice Guidelines of Nuclear Cardiology published by the Japanese Circulation Society included this procedure based on the considerable accumulation of clinical experience with ^123^I-MIBG in Japan [6, 7]. The European Association of Nuclear Medicine (EANM) Cardiovascular Committee and the European Council of Nuclear Cardiology have proposed MIBG protocols [8], and the American Society of Nuclear Cardiology (ASNC) imaging guidelines also summarize the application of ^123^I-MIBG and its methodology [9]. In addition to cardiology, ^123^I-MIBG has been used since the late 1990s with increasing frequency in patients with Parkinson’s disease and dementia with Lewy bodies, in whom cardiac ^123^I-MIBG uptake characteristically decreases due to neural degeneration [10, 11]. Thus, ^123^I-MIBG findings are considered as a biomarker of Lewy-body disease.

Although reproducibility of the heart-to-mediastinum ratio (HMR) is generally believed to be good [12], a major factor affecting HMR is differences among camera collimators at various hospitals [13]. For example, average normal values of late HMR are 2.5 with low-energy (LE) collimators and 3.0 for medium-energy (ME) collimators [14]. In fact, collimator designs are further divided into at least 6–7 collimator groups [15], and these differences are supposed to be mainly caused by different degrees of septal penetration and scatter in collimators, and the precise specifications of the size and length of holes and septal thickness are variable among vendors. We, therefore, developed a phantom-based correction method to cross-calibrate HMR among all Anger camera collimator systems [14]. Several phantom experiments have shown that even collimators of the same type, for example, low-energy high resolution (LEHR), have different specifications depending on the designs of vendors [17]. D-SPECT (Spectrum Dynamics, Israel; Biosensors Japan, Tokyo, Japan) has a cadmium–zinc–telluride (CZT) detector that enables high resolution and high sensitivity in myocardial perfusion imaging [17]. However, tomographic imaging is the standard output, and planar images commonly used with Anger cameras are not directly used. Differences between the Anger and D-SPECT cameras were investigated in the ADRECARD study, in which virtual anterior planograms were created with D-SPECT, and the HMR between the two methods correlated well [18].

The present study aimed to create a method of integrating HMR derived from D-SPECT planogram and Anger cameras using the same phantom-based conversion method to generate comparable quantitative parameters in ^123^I-MIBG study.

## Methods

### Phantom design and cross calibration of HMR

The structure of the calibration phantom is described elsewhere [14]. Briefly, the phantom was designed for planar imaging, and two reference HMR values can be obtained from one phantom using anterior and posterior sides (Hokuriku Yuuki, Co. Ltd., Kanazawa, Japan). Since the phantom has two compartments, one for ^123^I-MIBG and the other for water, the radionuclide concentration does not require adjustment, and HMR can be reproducibly calculated using dedicated software. The reference HMR values obtained from the anterior and posterior sides of the phantom were 2.6 and 3.5, respectively. The count decay in the acrylic and water compartment was calculated for thickness using an attenuation coefficient of 0.147/cm [19, 20].

A linear regression line that passes through a coordinate (1, 1) for the measured versus the reference HMR can be calculated, because two data points are obtained from the anterior and posterior sides. The slope of this regression line is defined as a conversion coefficient (CC) to the reference value, and it is unique for an institutional specific combination of scinticamera-collimator systems and acquisition conditions.

### Planograms generated by D-SPECT

The standard D-SPECT output comprised tomographic reconstructed images. Therefore, a planogram equivalent to a planar anterior image was created based on all elementary two-dimensional images that shared the same angle onto one large field of view in a virtual plane as described [18]. A series of two-dimensional images equivalent to those of SPECT with the Anger camera were obtained for every angular position. The phantom structure was designed for planar images, which render three-dimensional reconstruction meaningless. Therefore, we used only anterior planograms and repeated the acquisition on the reverse side of the phantom.

### Phantom experiments

**Fig. 1:**
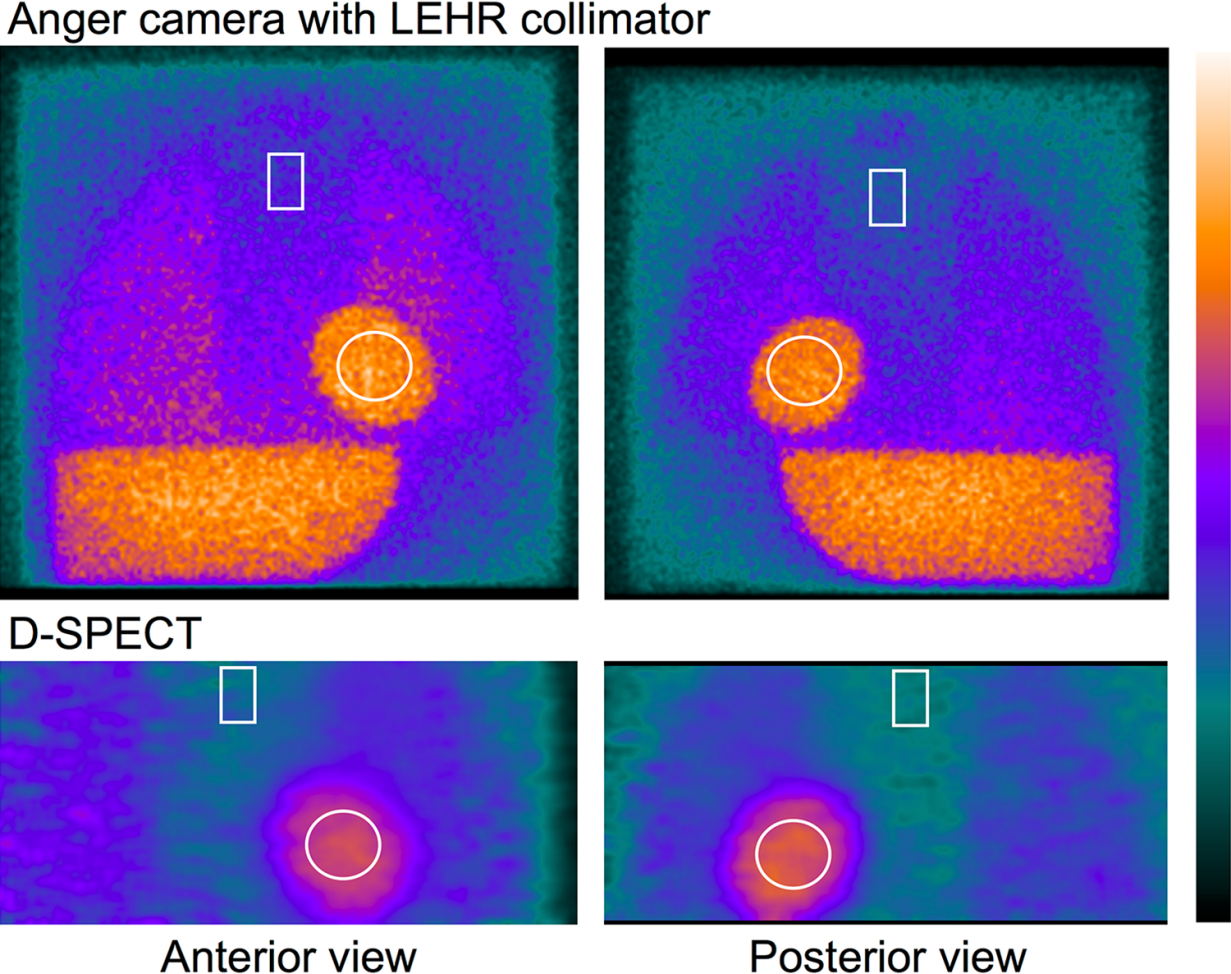
Phantom images acquired using Anger camera (**a**) and D-SPECT (**b**). Regions of interest are drawn on heart and mediastinum

Experiments with the calibration phantom proceeded for the Anger camera (Siemens Healthcare, Tokyo, Japan) with an LEHR collimator, and planar images were acquired from both sides of the phantom. Diluted ^123^I-MIBG (111 MBq) was poured into the phantom and images were acquired for 5 min each. A 15% energy window was set at 159 keV. Data were similarly acquired from both sides of the phantom for D-SPECT. The phantom was positioned horizontally on the backrest of an SPECT chair similar to how patients are positioned. A 3-cm acrylic plate filled with water was placed over the phantom when imaging was performed to simulate human body attenuation and scatter. A 15% asymmetric energy window was set at 159 keV (145–169 keV) for D-SPECT. Figure 1 shows typical phantom images.

### Clinical study

We retrospectively selected 40 consecutive outpatients (average age, 73 ± 10 years; male, 50%) referred for ^123^I-MIBG assessment between July 2016 and February 2017. The indications for ^123^I-MIBG assessment were determined by internal medicine physicians, and their aims for the study were to diagnose Parkinson’s disease and dementia with Lewy bodies for neurology, and to evaluate the diagnosis and prognosis of HF. The diagnosis of neurological diseases was made in 24 patients. Among the patients with HF, the average left ventricular ejection fraction ranged 20–70%.

We used data acquired from all enrolled patients, since our aim was to develop an optimal methodology. The Ethics Committee at the Public Central Hospital of Matto Ishikawa approved this research protocol. Written informed consent from individual patients was waived, because the MIBG studies comprised part of regular clinical practice without additional imaging.

Early and late images were acquired at 15 min and 3 h after an intravenous injection of 111 MBq of ^123^I-MIBG (MyoMIBG; FUJIFILM RI Pharma, Tokyo, Japan) using an Anger camera. The anterior images were obtained for 5 min each with a 256 × 256 matrix. Just after Anger camera images, D-SPECT images were acquired for 10 min. An anterior-view equivalent planograms were generated, and the HMR was then calculated. Tomographic imaging was a standard clinical procedure used for evaluating segmental defect caused by ischemia and extensive decrease in Lewy-body disease, but it was not used in the present study.

### Regions of interest and HMR

*Phantom analysis* We set regions of interest (ROI) on the heart and mediastinum of the phantom. The heart ROI was set as a circle on the heart, and the mediastinal ROI was set as a rectangle on the upper mediastinum with the Anger camera. The location was predefined for the phantom in all phantom experiments using the dedicated analytical software. Since the vertical image size was limited to 160 mm with D-SPECT, a similar ROI was manually set on the heart and mediastinum. The mediastinal ROI was set on the mid-mediastinum as high as possible, although lower than that in the Anger camera image.

*Patient analysis* The ROI was semi-automatically set as described for the clinical study (smartMIBG software, FUJIFILM RI Pharma, Japan) [21]. The operators selected a point at the center of the heart on the image, and then, a circular ROI was positioned on the heart. The subsequent processing was automatic, but can be modified manually if the location was inappropriate. A mediastinal ROI was determined as 30% of the height (center of the heart to the upper border of the mediastinum) and 10% of the body width. The circular cardiac ROI was similar to the setting in the study using the Anger camera. Since an upper mediastinum ROI could not be set for D-SPECT imaging, the highest mediastinal region of the lowest average count was selected. In a preliminary study, the inter-observer reproducibility of average mediastinal counts in the initial 40 data points was good, showing the first measurement = 1.01 × the second measurement −8 (*r* = 0.99, *p* < 0.0001).

### Conversion of HMR between Anger camera and D-SPECT

To adjust HMR from D-SPECT to Anger camera conditions, the following equation was used:

Adjusted HMR = CC of Anger camera/CC of D-SPECT × (measured HMR − 1) + 1, and the effect of correction was examined.

In the next step, we used an MEGP collimator condition to standardize the HMR to provide better quantitative accuracy as stated in the European imaging proposal [8]. The average CC with MEGP was 0.88 [15], which is referred to herein as “standard ME88”. Based on the measured CC for the system, the standardized HMR to the ME88 condition was calculated as:

Standardized HMR = 0.88/CC of the institutional system × (measured HMR − 1) + 1.

### Calculation of washout rate

Washout rate (WR) was calculated using the following formula for both the Anger camera and D-SPECT with early and late heart counts (*H*_early_, *H*_late_), mediastinal counts (*M*_early_, *M*_late_), and a decay correction factor (DCF):

$${\text{WR}} = ((H_{\text{early}} - M_{\text{early}} ) - (H_{\text{late}} - M_{\text{late}} )/{\text{DCF}})/(H_{\text{early}} - M_{\text{early}} ) \times 100,$$

$${\text{WR without background correction}} = (H_{\text{early}} - H_{\text{late}} /{\text{DCF}})/H_{\text{early}} \times 100,$$

where DCF = 0.5^(difference between early and late (h)/13).

### Statistics

Data are shown as mean ± standard deviation (SD). Differences among groups were assessed using the one-way analysis of variance and Student’s *t* test. Paired values were analyzed using paired *t* tests with Bland–Altman plots and signed rank tests. Linear regression of the HMR between the two camera conditions was calculated using the least squares method. A variability of the average ROI count was also examined using coefficient of variation (CV, %). The statistics software was JMP version 12 (SAS Institute Inc., Cary, NC, USA), and we used Mathematica 11 (Wolfram Research Inc., Champaign, IL, USA) for some of the mathematical calculations.

## Results

### Phantom experiments and conversion coefficients

Figure 1 shows phantom images obtained with the Anger camera and D-SPECT planograms. Based on the two measurements, CC was calculated as 0.55 for the Anger camera with an LEHR collimator and 0.63 for the D-SPECT camera.

### Cardiac and mediastinal counts

**Fig. 2:**
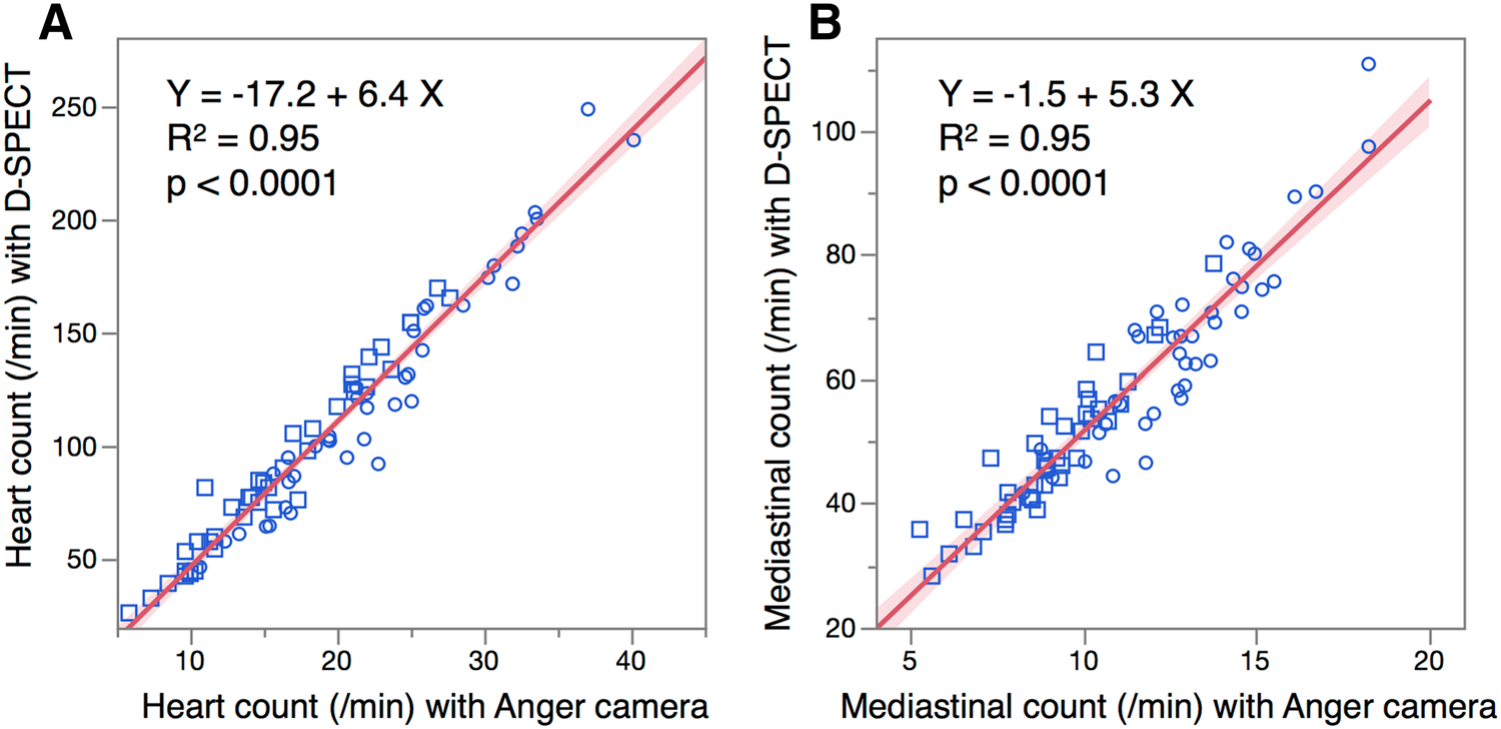
Relationship of cardiac (**a**) and mediastinal (**b**) counts/pixel per minute derived from Anger camera and D-SPECT. *Circles* and *squares* early and late HMR, respectively. *Shaded area* confidence of fit

Cardiac and mediastinal counts per minute were compared between the Anger and D-SPECT cameras (Fig. 2). Linear correlation was good for both cardiac and mediastinal counts (*R* ^2^ = 0.95 for both), whereas acquired counts were higher with D-SPECT than the Anger camera. Mediastinal count variability was similar in two groups with lower (HMR < 1.6) and upper (HMR > 2.8) quartiles of HMR distribution. In the lower quartile group, mean count/pixel/min and CV (%) were 10.2 (22%) for Anger camera and 50.8 (23%) for D-SPECT. In the upper quartile group, they were 12.0 (24%) for Anger camera and 62.6 (25%) for D-SPECT.

### Cross calibration of HMR ratio in the clinical study

**Fig. 3:**
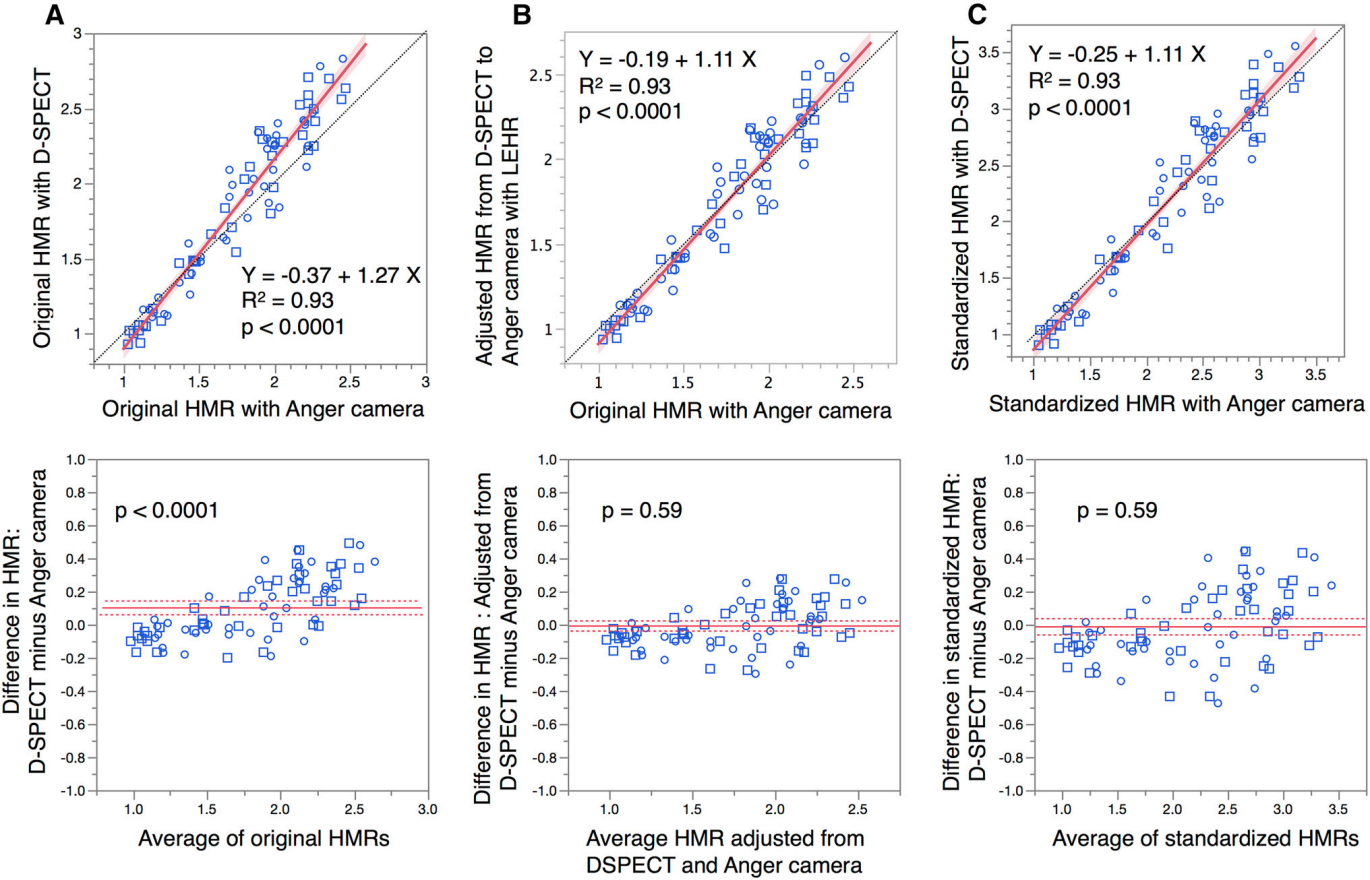
HMR derived from Anger camera and D-SPECT. Original HMR (**a**), HMR adjusted from D-SPECT to Anger camera condition (**b**), and HMR standardized to ME 88 condition (conversion coefficient of 0.88 with MEGP collimator) (**c**) are shown. HMR derived from Anger camera and D-SPECT showed systematic error when HMR is high (HMR is higher from D-SPECT than Anger camera), as shown in Brand–Altman plot (*p* < 0.0001) (**a**). Adjustment of HMR to LEHR collimator condition or ME88 condition improved correspondence between both HMR (p = n. s. for both). *Circles* and *squares* early and late HMR, respectively. *Dotted line* line of identity. *Shaded area* confidence of fit. *Solid line* in pairwise comparison plot, mean difference; *dotted lines* upper and lower 95% of mean difference

Table 1 shows the HMR derived from the original Anger and D-SPECT images. Paired comparisons of the HMR derived from the original Anger and D-SPECT images showed that the latter was significantly higher (*p* < 0.0001), with a mean difference of 0.10. When HMR from D-SPECT was converted to the condition of the Anger camera with LEHR collimator, the difference between two systems disappeared (p = n. s.). Scatterplots and linear regression lines between HMR before and after correction showed that the conversion of HMR from D-SPECT to the Anger camera condition improved the systematic differences between the two camera systems (Fig. 3a, b).

**Table 1:** Original and standardized heart-to-mediastinum ratio

Camera-Collimator and CC	Original HMR—Anger camera	Original HMR—D-SPECT	D-SPECT adjusted to Anger LEHR condition	Anger LEHR standardized to ME88 condition	D-SPECT standardized to ME88 condition
	Anger LEHR (CC = 0.55)	D-SPECT (CC = 0.63)	D-SPECT (CC = 0.63) to Anger LEHR (CC = 0.55) conditions	Anger LEHR (CC = 0.55) to Anger MEGP (CC = 0.88) conditions	D-SPECT (CC = 0.63) to Anger MEGP (CC = 0.88) conditions
Mean	1.76	1.86	1.75	2.21	2.20
SD	0.40	0.54	0.48	0.65	0.75
Minimum	1.03	0.93	0.94	1.05	0.90
Maximum	2.47	2.83	2.60	3.35	3.56
Analysis versus	–	Original HMR—Anger	Original HMR—Anger	–	Anger LEHR standardized to ME88
Mean difference		0.10	0.01		0.01
*P*		<0.0001	0.59		0.59
Wilcoxon signed rank		<0.0001	0.52		0.52
Correlation *R*		0.96	0.96		0.96

*CC* conversion coefficient, *HMR* heart-to-mediastinum ratio, *LEHR* low-energy high-resolution collimator, *ME88* ME collimator with conversion coefficient of 0.88, *SD* standard deviation

Standardization to the ME88 condition similarly eliminated the difference between the Anger and D-SPECT findings. The average standardized HMR from the Anger camera (StdHMR_Anger_) and D-SPECT (StdHMR_DSPECT_) became comparable (2.21 ± 0.65 vs. 2.20 ± 0.75, p = n. s.) (Table 1). After standardization, a bivariate correlation plot showed good linearity: StdHMR_DSPECT_ = −0.25 + 1.11 × StdHMR_Anger_ (*R* ^2^ = 0.93; Fig. 3c).

### Additional correction of HMR

**Fig. 4:**
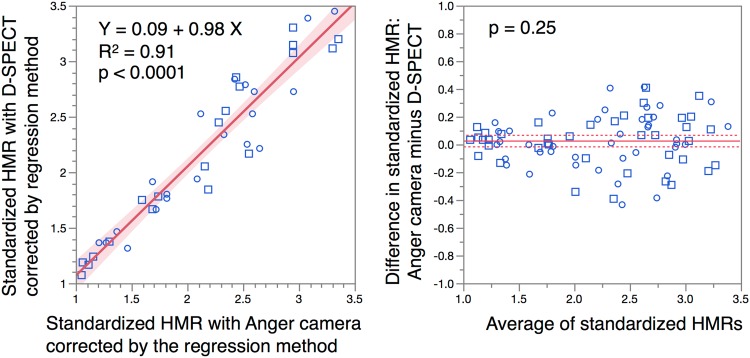
Additional correction of HMR in the latter 40 data points using a regression line derived from the initial 40 data points. A slight deviation of the line observed in Fig. 3c was further improved. *Shaded area* confidence of fit. *Solid line* in pairwise comparison plot, mean difference; *dotted lines* upper and lower 95% of mean difference

Since standardized HMR_DPSECT_ was slightly lower in the range of HMR < 1.3 and slightly higher in the range of HMR > 2.3 (Fig. 3c) compared with the standardized HMR_Anger_, further correction was attempted. As standardized HMR with Anger camera was calculated as StdHMR_Anger_ = (StdHMR_DSPECT_ + 0.30)/1.19 in the initial 40 data points, this regression equation was applied to the latter 40 data points for validation. Then, the bivariate correlation plot showed improved linearity: StdHMR_DSPECT_ = 0.09 + 0.98 × StdHMR_Anger_ (*R* ^2^ = 0.91; Fig. 4).

### Washout rates

**Fig. 5:**
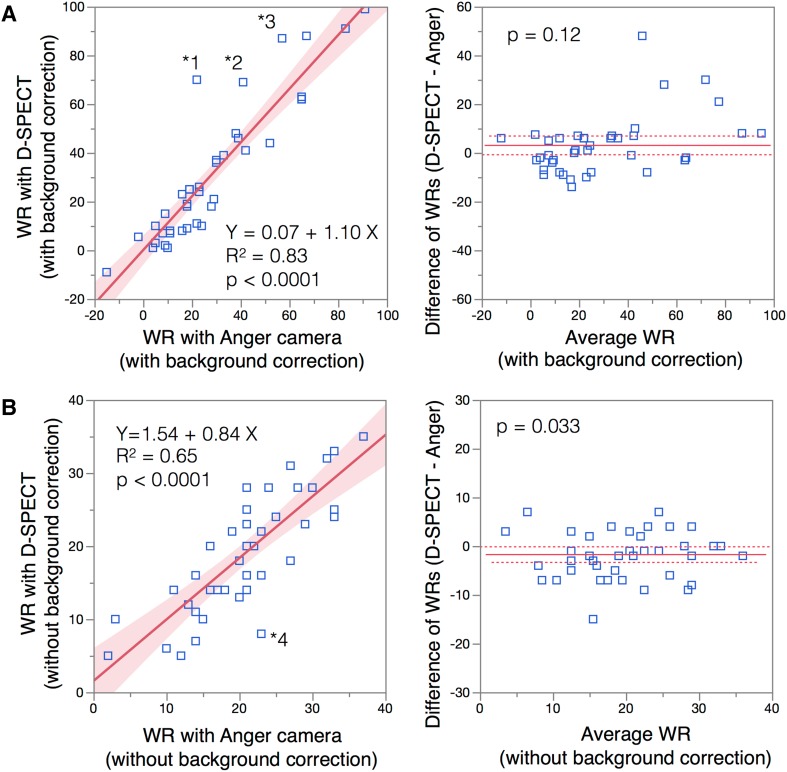
Correlation of washout rates (WR) derived from D-SPECT and Anger camera with (**a**) and without (**b**) background subtraction. *Asterisk* four outlier data points (indicated as 1–4) were from patients with HMR ≤ 1.1 (1.08, 1.10, 1.10, and 0.92, respectively), who had low cardiac and background counts. *Shaded area* confidence of fit. *Solid line* in pairwise comparison plot, mean difference; *dotted lines* upper and lower 95% of mean difference

Washout rates from the D-SPECT and Anger cameras were compared with and without background correction (Fig. 5). Although they positively correlated (*R* ^2^ = 0.83 and 0.65, *p* < 0.0001, respectively), a few outliers persisted between the values derived from both cameras.

## Discussion

The major purpose of this study was to create a conversion method between Anger and D-SPECT CZT cameras. Using CC values for D-SPECT image acquisition, we cross-calibrated HMR between Anger and D-SPECT camera systems and could also adjust the HMR to the ME88 condition. This cross calibration could enable the application of HMR to multicenter studies of patients with chronic HF and Lewy-body disease.

### Need for standardization of MIBG parameters

Although HMR in a ^123^I-MIBG study is a simple parameter based on the average count ratio of the heart and mediastinum, a standardized approach is essential for diagnostic and prognostic evaluation [22]. Among various factors, the influence of the collimator on HMR calculations is too large to generate consistent results, particularly when the collimators are of low-(LE) and medium-(ME) energy. Several methods have been proposed, but we advocate using a calibration phantom that can be easily applied to any camera-collimator setting [15, 23]. The findings of 225 phantom experiments have shown that the key characteristics of collimators are not simply ME and LE, but can be more precisely defined, for example, as LEHR, LE general-purpose (LEGP), low–medium energy (LME), MEGP, and ME–low penetration (MELP) [15]. Whether or not a CC could be similarly determined for D-SPECT after the advent of the CZT camera has remained unknown. Even if CC could be measured, whether or not a D-SPECT HMR could be integrated with Anger camera conditions has not been determined.

### Characteristics of HMR by D-SPECT

Anterior planar image-equivalent planograms generated by D-SPECT can serve as part of a quality control system for projection images, which was a convenient base for this study. Although we tried similar ROI settings, D-SPECT has some limitations. An upper mediastinal ROI cannot be set due to the vertical length of the view being 16 cm. We, therefore, tried to define the highest possible mediastinal region with the lowest average count, which corresponded to the mid position on the Anger camera image. If the large field of view CZT camera is available in future, the effect of small field of view on the accuracy of ROI setting could be validated. In addition, mediastinal count variability as examined by CV was not significantly different between the Anger camera and D-SPECT in patients with high and low HMR. However, an automatic ROI processing algorithm for D-SPECT could enhance reproducibility. The energy resolution of the CZT camera is better, which enabled better contrast in images derived from D-SPECT than from the Anger camera. The Compton scatter fraction might also differ between Anger and D-SPECT settings. Therefore, the CC determined in this study is a practical value with which to cross-calibrate the two camera conditions. The planogram is unique to the single D-SPECT system, whereas images from the Anger camera vary due to wide disparities among camera-collimator combinations.

Although the administration dose of ^123^I-MIBG was relatively low (111 MBq) compared with studies in the North America (370 MBq) and Europe (185 MBq), the image quality of planogram and SPECT was good by 10-min acquisition.

### Comparison with ADRECARD study

The ADRECARD study was the first to compare HMR calibrated using D-SPECT and Anger cameras. A conversion equation for HMR (Corrected D-SPECT) = 0.5896 × HMR (D-SPECT) + 0.4649 was created based on a phantom experiment in that study [18]. Based on their original table and assuming that the LEHR of their camera collimator had a CC of 0.55, we tentatively calculated the standardized HMR, and found a good correlation even with our standardization method. However, HMR (D-SPECT) + 0.1 seemed to correlate more closely with the standardized HMR (Anger) [24]. Our HMR calculated with the Anger camera was higher than that in the ADRECARD study. Although agreement with our temporary calculation was generally good in the present study, the following factors should be considered. To calculate HMR, the square ROI over the heart applied in the ADRECARD study included a slight extra-cardiac area (lower heart count than ours), and a mediastinal rectangular region was placed in a lower position (higher mediastinal count than ours). Our heart ROI setting in the clinical study was circular and within the heart, and we identified the mediastinal region with the lowest count. As a result, the HMR calculated from the Anger camera using our algorithm was always higher. The location of the ROI could be a cause of variation in the clinical setting [25]. Although the ADRECARD study used ^99m^Tc-tetrofosmin for localization of the heart, we did not use dual-nuclide acquisition to reduce the radiation burden and study cost. We obtained anterior planograms by localizing the heart by pre-test imaging for a short period. When the field of view is inappropriately located, measurements could be readily repeated using the high-sensitivity D-SPECT system.

### Clinical implications

The difference in measured HMR between D-SPECT and Anger camera with an LEHR collimator was smaller compared with that between the Anger camera with LEHR and ME collimators. The CC with D-SPECT was between that of LEHR and LME collimators [15, 20, 23]. In multicenter prognostic studies, HMR values of 1.6–1.75 were thresholds for differentiating good and poor prognosis including cardiac death, serious arrhythmia, and progression of HF [1, 2, 4, 7]. When the linear regression line was observed, the impact of cross calibration was relatively small in the HMR range of <1.6. However, in the range of borderline to higher HMR, the discrepancy was increased between the two systems and appropriate correction methods should be used.

In a D-SPECT study, conversion of HMR to ME88 condition using conversion coefficient (0.63) works well around the HMR of 1.6–2.0 and can be used for clinical studies for differentiating good and poor prognosis. However, standardized HMR with D-SPECT showed slightly lower values in the HMR range of <1.3 and higher values in the HMR range of >2.3. This was probably due to lower mediastinal background and better contrast in D-SPECT study compared with the Anger camera condition. This systematic difference could be further corrected if we used regression line between standardized values. However, as the need for additional correction may depend on the individual D-SPECT system and acquisition conditions, further studies should be indicated in multiple centers, where D-SPECT is used.

If HMR could be consistently calculated in a wide range of HMR, possibility of using D-SPECT for a mortality risk model could be considered [26]. Since the uncorrected D-SPECT HMR was higher than that of the Anger camera with an LE collimator in the borderline to higher HMR range, a corrected HMR could avoid underestimating mortality risk, although further validation studies will be required.

Although the LEHR collimator is popular in the United States, many types of collimators other than LEHR such as LEGP, LME, MEGP, and MELP collimators are actually used in clinical practice. Therefore, CC can be applied to compare HMR from various conditions using the uniform acquisition conditions, such as ME88 condition and individual institutional LE collimator condition.

### Washout rate

While the correlation between washout rates derived from the Anger camera and D-SPECT was also fair, reproducibility requires careful attention when background subtraction is applied to very low cardiac counts as seen in the outliers of washout rate plots between both cameras (Fig. 5). The need for background correction when patients have a low HMR should be further analyzed from both diagnostic and prognostic viewpoints [12].

### Limitations

Only one D-SPECT and one Anger camera system were included in this study. Although D-SPECT planograms have no potential for variation, the adequacy of applying the present results to other hospitals should be further studied. We had already completed phantom experiments under 225 conditions at 84 institutions [15, 27] (>1000 conditions at present) in Japan by the end of 2016 and by that time studies under 210 conditions had also proceeded at 27 European institutions [20]. Although CC values are affected by specifications of the camera, collimators, detector crystals, and acquisition conditions, we postulate that the present findings could be applicable even for D-SPECT compared with other camera-collimator combinations. However, further studies are needed to validate this phantom methodology for universal applications. Three-dimensional SPECT quantitation was not used in the present study. Because whole heart quantitation has been achieved using MIBG imaging [28, 29], the potential variability of such three-dimensional methods including the need for an appropriate background, dependency of the results on software algorithm, and the relationship to conventional planar imaging should be further investigated. Whereas the current study confirmed that planar-equivalent HMR can be generated from D-SPECT images, whether this is the optimal use of the imaging capabilities of this D-SPECT system remains to be determined. Finally, in the clinical application, timing of the Anger and D-SPECT was not exactly the same. However, correlation of average counts was good between the Anger and D-SPECT cameras, and it has been shown that variation in acquisition time of ^123^I-MIBG between 2- and 4-h post-injection did not lead to a clinically significant change in the late *H*/*M* ratio [30].

## Conclusion

The ^123^I-MIBG HMR can be similarly calculated with D-SPECT using a planogram as used in planar studies with an Anger camera. The HMR derived from D-SPECT can be calibrated to both LE collimator and ME collimator conditions using CC values based on institutional phantom experiments. A slight deviation of the regression line could be further improved using the regression line. The cross-calibration method supports diagnostic and prognostic uses of D-SPECT as used in Anger camera systems.

## Abbreviations

CC: Conversion coefficient
CZT: Cadmium–zinc–telluride
HF: Heart failure
HMR: Heart-to-mediastinum ratio
LE: Low energy
ME: Medium energy
ME88: Medium-energy collimator condition with a conversion coefficient of 0.88
MIBG: *Meta*-iodobenzylguanidine
ROI: Region of interest
